# Antibiotic Susceptibility Testing with Raman Biosensing

**DOI:** 10.3390/antibiotics11121812

**Published:** 2022-12-14

**Authors:** Andrei Novikov, Adeliya Sayfutdinova, Ekaterina Botchkova, Dmitry Kopitsyn, Rawil Fakhrullin

**Affiliations:** 1 Department of Physical and Colloid Chemistry, Gubkin University, 65/1 Leninsky Prospect, 119991 Moscow, Russia; 2 Institute of Fundamental Medicine and Biology, Kazan Federal University, 420008 Kazan, Republic of Tatarstan, Russia

**Keywords:** bacteria, antibiotics, nanoparticles, nanomaterials, surface-enhanced Raman scattering, antibiotic susceptibility, multidrug resistance, minimum inhibitory concentration

## Abstract

Antibiotics guard us against bacterial infections and are among the most commonly used medicines. The immediate consequence of their large-scale production and prescription is the development of antibiotic resistance. Therefore, rapid detection of antibiotic susceptibility is required for efficient antimicrobial therapy. One of the promising methods for rapid antibiotic susceptibility testing is Raman spectroscopy. Raman spectroscopy combines fast and contactless acquisition of spectra with good selectivity towards bacterial cells. The antibiotic-induced changes in bacterial cell physiology are detected as distinct features in Raman spectra and can be associated with antibiotic susceptibility. Therefore, the Raman-based approach may be beneficial in designing therapy against multidrug-resistant infections. The surface-enhanced Raman spectroscopy (SERS) and resonance Raman spectroscopy (RRS) additionally provide excellent sensitivity. In this review, we present an analysis of the Raman spectroscopy–based optical biosensing approaches aimed at antibiotic susceptibility testing.

## 1. Introduction

Antibiotics remain the most essential drugs in the treatment of bacterial infections [1]. Recently, more and more studies have shown that the emergence of “superbugs” could combine the resistance to all known antibiotics, thus making available treatments ineffective [2,3]. The rapid increase of antibiotic resistance in microorganisms is believed to be caused by antibiotic pollution from industrial and hospital effluents [4,5,6]. Furthermore, hospital conditions where both disinfectants and antibiotics are used provide a low-competitive ecological niche for multidrug-resistant bacteria [7]. Another complicating factor for multidrug resistance is the widespread systemic antibiotic therapy used during the COVID-19 pandemic, associated with frequent bacterial co-infection [8]. In particular, the COVID-19 pandemic–related increase of antibiotic consumption necessitates a careful antibiotic stewardship in the post-pandemic period for preventing irreversible antimicrobial prevalence [9]. This suggests that effective methods to monitor antibiotic effects and resistance in microorganisms are required.

Surface-enhanced Raman spectroscopy (SERS) is a powerful analytical method having single-molecule sensitivity [10,11]. As a result, SERS has found numerous applications in biomedical research. A simple and efficient strategy for SERS substrate preparation is the aggregation of gold or silver nanoparticles [12,13], with an optional oxidation or electrochemical removal of capping agents for additional signal improvement [14,15]. A recent study revealed the dynamic nature of SERS signal generation in nanoparticle-based substrates [16]. Another notable effort is the development of microwell plate-based Raman readers [17,18] for high-throughput chemical screening.

The practical applications of SERS include detection of various trace chemicals, such as pesticides [19,20], drugs [21,22,23] or disease biomarkers in blood [24]. Antibiotics can also be directly detected by SERS, as was summarized in the recent reviews [25,26]. Biological samples (e.g., viruses and bacteria) can be analyzed after proper purification but often require complicated spectra processing for interpretation [27,28,29,30], as the bacterial SERS spectra are not only highly similar but dependent on many factors, with a significant contribution from excreted purine moieties [31]. Indirect SERS-based approaches can be employed for bacterial colonization monitoring [32] and cytotoxicity evaluation [33]. Furthermore, gold–silver nanoparticles may serve as bactericidal agents and SERS substrates, simultaneously providing antimicrobial action and antibiotic-resistant bacteria detection [34].

In this review, we present an analysis of the Raman spectroscopy–based optical biosensing approaches aimed at antibiotic susceptibility testing and prospective antibiotic detection.

## 2. Antibiotic Susceptibility

### 2.1. Antibiotics and Raman Sensing

**Figure 1:**
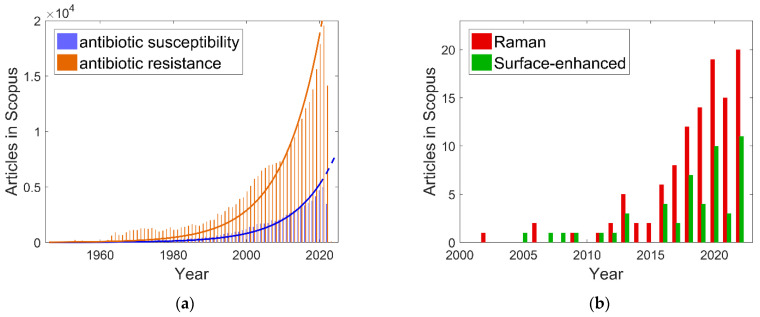
The number of papers indexed in Scopus per year: (**a**) queries “antibiotic AND susceptibility” (blue bars; the blue line is an exponential approximation) and “antibiotic AND resistance” (orange bars; the orange line is an exponential approximation); (**b**) queries “antibiotic AND susceptibility AND Raman” (red bars) and “antibiotic AND susceptibility AND surface-enhanced” (green bars).

Antibiotic susceptibility studies have been published since 1945, when Neter found that various strains of *Staphylococci* have different susceptibility to available antibiotics [35]. The number of published articles in this area has grown exponentially in the last decade (Figure 1a). Similarly, antibiotic resistance has been a steady concern, with more than one hundred papers published annually since 1962. In the last decade, published articles on “antibiotic resistance” have increased greatly, with roughly 3.57 times more papers published on this topic than on “antibiotic susceptibility”.

Active bacterial cells differ from resting cells by certain features in their Raman spectra, as was noted by Webb and Stoneham in 1977 [36]. Resonance Raman spectroscopy of carotene-containing bacteria and algae was employed for their identification in 1980 [37]. However, bacterial constituents are weak Raman scatterers, and the collection of well-detailed bacterial Raman spectra is rather difficult. The invention of surface-enhanced Raman spectroscopy [38] opened the way for the convenient registration of bacterial spectra, which was demonstrated in the pioneering works of Efrima et al. [39,40,41] and Goodacre et al. [42,43]. Since then, Raman spectroscopy has emerged as a fast and informative technique for studying the antibiotic susceptibility (Figure 1b). Note that only 31 papers were found in Scopus with the query “{antibiotic susceptibility} Raman AND NOT surface-enhanced” (not shown in Figure 1). Most of the studies employ surface-enhanced Raman spectroscopy.

### 2.2. Antibiotic Action on Bacteria and Mechanisms of Their Resistance

**Figure 2:**
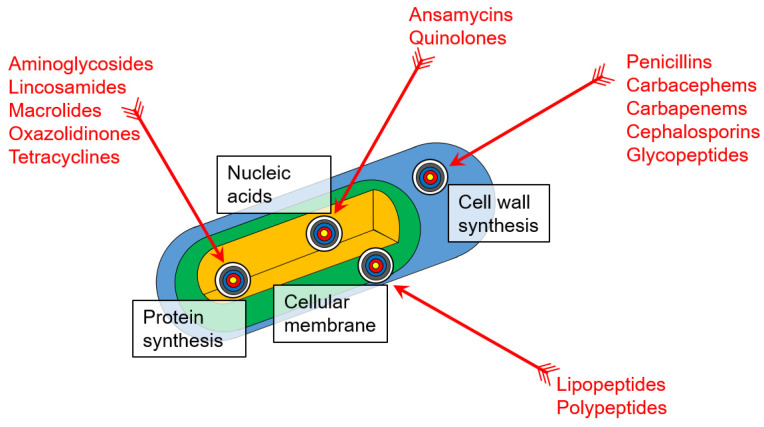
Scheme demonstrating the mechanisms of antibiotic action on bacteria.

**Figure 3:**
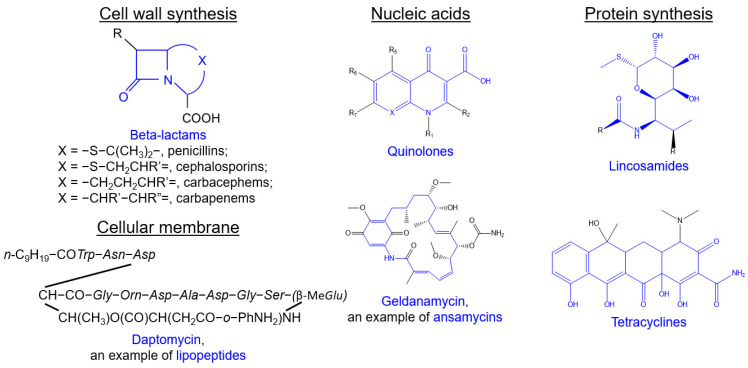
The chemical structures of various antibiotic classes juxtaposed with their biochemical target names (the structural motifs typical for the antibiotic classes are shown in blue).

Antibiotics are strikingly efficient against bacteria because of the significant differences in the biochemistry of prokaryotic and eukaryotic cells. The common biochemical targets of antibiotics are shown in Figure 2, and several structures of various classes of antibiotics are shown in Figure 3. Here, we will briefly review the common antibiotic classes, their mechanism of action, bacterial resistance strategies, and the recent approaches to overcome this resistance.

Bacteria develop resistance to antibiotics due to spontaneous genetic mutations. The key process involved is the horizontal gene transfer, i.e., transfer of mobile genetic elements, such as integrons and plasmids, that contain antibiotic-resistant genes [44]. This process is particularly effective in multi-species biofilms [45]. Various exogenous factors can favor the acquisition of antibiotic resistance, such as inappropriate prescription of antibiotics, self-medication, excessive use of antibiotics in agriculture, etc. Another important factor stimulating the distribution of antibiotic resistance is the selective pressure caused by antibiotic pollution, lack or absence of wastewater treatment, and bioremediation in soils [4,46].

#### 2.2.1. Beta-Lactams

Several important antibiotic classes disrupt the microbial cell wall biosynthesis. Beta-lactam antibiotics (such as penicillins, carbapenems, or cephalosporins) have structural similarity to the D-Ala-D-Ala fragment of the peptidoglycan building blocks. Highly reactive carbonyl in the β-lactam system ensures the acetylation of the serine residue in the active center of transpeptidases involved in the final steps of the peptidoglycan synthesis [47]. The widespread use of the β-lactams induced the arms race between medicinal chemists and bacteria. The emergence of β-lactamases containing active serine residue of zinc-based active centers and their dissemination via horizontal gene transfer led to the bacterial resistance to many commercial β-lactam antibiotics. Thus, the modern β-lactams are often prescribed with the β-lactamase inhibitors (such as clavulanic acid), aiming to irreversibly bind to the active center of enzymes and render them inactive [48]. Another complication of β-lactam usage is the possible large-scale destruction of intestinal microbiota with a consequent “peptidoglycan storm”, the massive release of aminoglycoside residues provoking the pathogenic growth of some fungi [49].

#### 2.2.2. Glycopeptides

The resistance to β-lactams drove the development of glycopeptide antibiotics (such as vancomycin) that bind to the terminal D-Ala-D-Ala fragment and thus disrupt the proper synthesis of peptidoglycans [50]. Resistance to vancomycin is accomplished via hydrolysis of dipeptide D-Ala-D-Ala peptidoglycan precursors and further synthesis of peptidoglycan precursors with low affinity to vancomycin. In such precursors, the C-terminal D-Ala residue is replaced by D-lactate (D-Lac) or D-serine (D-Ser). Another strategy is connected with the elimination of the high-affinity precursors that are normally produced by the host, thus removing the vancomycin-binding target [51,52]. In recent years, several modifications of the core glycopeptide structure were proposed that add complementary inhibiting mechanisms and overcome antibiotic resistance [53,54].

#### 2.2.3. Polypeptides

Several polypeptide antibiotics (such as polymyxins and gramicidins), being structurally similar to glycopeptides, target bacterial membranes. As one may expect, these antibiotics are less selective than their counterparts targeting cell wall biosynthesis, and have considerable toxicity [55,56]. Gramicidins form transmembrane dimers that serve as ion channels, thus increasing the membrane permeability and killing prokaryotic and eukaryotic cells [57,58]. Microbial resistance to polypeptide antibiotics can be based on various strategies: modifications in lipopolysaccharide structure (lipid A in particular, which is the basic target site for this group of antibiotics), the use of efflux pumps, formation of capsules, and overexpression of the outer membrane proteins [59]. Recently, the less hydrophobic polymyxin derivatives were proposed, which have reduced toxicity and effectiveness but still bind to the bacterial lipopolysaccharide and contribute to the antibacterial action by increasing the membrane permeability of Gram-negative bacteria [60]. The structure of dimer-forming gramicidins can be adjusted by disulfide bonding, thus fine-tuning their bioactivity [61].

#### 2.2.4. Lipopeptides

The lipopeptide antibiotic daptomycin has attracted much attention due to its unusual mode of action. As shown by combined proteomic and fluorescent lipid probe studies, daptomycin affects bacterial membrane fluidity, consequently blocking cell wall synthesis and altering phospholipid synthesis, and thus affecting respiration, membrane potential, and cell division [62]. This intricate mechanism of action explains the remarkable selectivity of daptomycin towards prokaryotes [63]. The resistance to daptomycin, still poorly understood, is accomplished through various mechanisms of action. Some environmental, non-pathogenic organisms harbor enzymes that hydrolyze and inactivate daptomycin [64]. However, pathogens use other ways to resist daptomycin action, e.g., by mutations in genes associated with cell wall synthesis, changes in cell membrane charge, and phospholipid metabolism [64,65].

#### 2.2.5. Quinolones

Prokaryotic nucleic acids are another obvious target for antibiotics. The topology of circular bacterial DNA is controlled by the crucial type II topoisomerases: gyrase and topoisomerase IV. Quinolones, synthetic antibiotics introduced in clinical practice about five decades ago, exploit these enzymes for targeting the bacterial DNA, forming non-covalent complexes at the enzyme–DNA interface and converting type II topoisomerases into cellular poisons [66]. Resistance to quinolones may be caused by the mutations in the target enzymes, chromosome-mediated regulation of the transmembrane transport, and plasmid-mediated synthesis of the defensive proteins [67]. The first mechanism is the most frequent; it is connected with mutations in gyrase- and topoisomerase IV-coding genes, thus leading to decreased binding of antibiotics with enzymes. Therefore, topoisomerase is losing its ability to inhibit DNA ligation or to form stable ternary complexes [68]. Thus, the obvious strategy to overcome this resistance is the design of quinolone derivatives or analogs (novel bacterial topoisomerase inhibitors) that would bind to the type II topoisomerases by a different mechanism [69]. However, efflux-mediated resistance (achieved by overexpression of transport genes) to quinolones has also become a common resistance mechanism [67].

#### 2.2.6. Aminoglycosides

Bacterial protein synthesis machinery differs from its eukaryotic counterpart, making it a target for several types of drugs. Aminoglycoside antibiotics bind into or close to the A-site in the 16S rRNA (with an important exception of streptomycin, which disrupts the interaction of 16S rRNA with tRNA), causing misreading of mRNA, leading to synthesis of faulty proteins [70]. The principal resistance mechanisms involve aminoglycoside-modifying enzymes, target modifications, and regulation of aminoglycoside transport [71]. Defensive enzymes can modify hydroxyl and amine groups of aminoglycosides with acetyl, phosphoryl, or nucleotidyl moieties. Although the A-site of 16S rRNA is highly conserved and its mutations are lethal, target modifications may include mutations of ribosomal proteins or methylation of 16S rRNA. Transport of aminoglycosides may be disrupted by the alterations in lipopolysaccharide or by efflux pumps. However, these mechanisms are metabolically costly, and aminoglycoside-modifying enzymes remain the primary concern. Numerous strategies for overcoming the resistance were explored, including, e.g., alkyl/aryl substitution, aminoglycoside core modification, and conjugation [72].

#### 2.2.7. Macrolides

The functioning of bacterial ribosomes can also be affected by the macrolides—small molecules inducing dissociation of the peptidyl-tRNA complex from ribosome and the premature interruption of the protein synthesis [73]. The resistance to macrolides can be based on rRNA methylation and acquisition of the defensive macrolide-modifying enzymes and transporter enzymes [74].

#### 2.2.8. Tetracyclines

Tetracyclines bind to RNA, including bacterial rRNA. Although the precise mechanism of action remains elusive, the broad activity spectrum and correlation of 16S rRNA mutations with tetracycline resistance suggest the direct binding to 16S rRNA [75]. Recent studies show that the enzymatic inactivation of tetracyclines could be overcome by suitable inhibitors [76]. However, the most common mechanism of tetracycline inactivation is based on an active efflux facilitated by efflux proteins or with ribosomal protection [77,78].

### 2.3. Raman Spectroscopy and Raman Spectra of Bacteria

**Figure 4:**
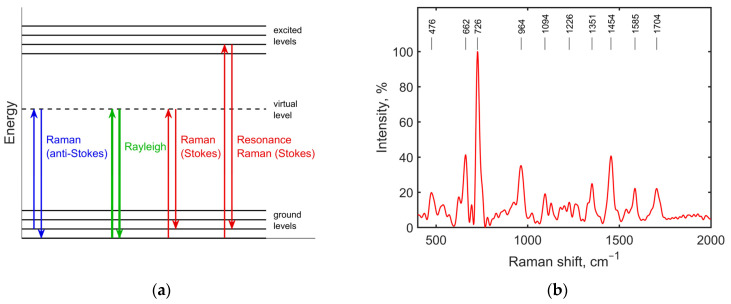
Mechanism of Raman scattering (**a**) and an example of *E. coli* SERS spectrum (**b**) with 785-nm excitation (replotted using data from [29]).

Inelastic light scattering, occurring as a result of the interaction of photons with vibrations of atoms or molecules, was predicted by Smekal [79] and then observed by Raman and Krishnan in various liquids [80] and by Mandelstam and Landsberg in quartz [81]. The comprehensive history of Raman scattering development is summarized in the book by Cardona and Merlin [82]. The energy diagram illustrating various scattering mechanisms is shown in Figure 4a. Low intensity of the normal Raman (NR) signal encourages the use of special Raman effects: resonance and surface-enhanced Raman scattering. Resonance Raman scattering (RRS) occurs when exciting photons match the energy gap between the ground and excited state (i.e., the excitation wavelength matches the absorbance band) [83]. Surface-enhanced Raman scattering occurs when the molecule of interest is near the roughened metal surface, with a surface plasmon resonance band overlapping excitation and emission wavelengths [38,84,85]. After the first observations of intensive resonance Raman spectra of carotenoids in living plant tissue [86], registration of the resonance [37] and normal Raman spectra of living bacterial and mammalian cells soon followed [87]. An example of the *E. coli* SERS spectrum is shown in Figure 4b.

### 2.4. Features in the Raman Spectra of Bacteria Affected by Antibiotics

The key question of using Raman spectroscopy is whether there are significant differences between the spectra of susceptible/resistant bacteria treated/untreated with antibiotics. In other words, are there any features in Raman spectra that can be exploited as biomarker signals to identify the effect of antibiotics on bacteria?

The Raman spectra of bacteria are strongly affected by laser wavelength, bacterial species, growth phase of bacteria, substrate, etc., and the interpretation of spectra is a challenging task. The proliferation state of bacteria and its susceptibility to antibiotics can be identified by observing dynamic changes occurring in the SERS spectra of living bacteria. In addition, the effect of antibiotics and/or antibiotic susceptibility can be recognized by the altered spectra of metabolites secreted by bacteria. Therefore, there are two approaches to study the antibiotic effect on bacteria—direct investigation of bacterial cells (either in suspension or on the solid support) and detection of secreted metabolites (for example, by studying the extracellular matrix) [88].

The purine derivatives adenine and hypoxanthine released from common pathogens *S. aureus*, *E. coli*, *P. aeruginosa*, *S. pneumonia,* etc., can be effectively detected by Raman spectra [89]. The Raman shifts of both are found in the range of 726–740 cm^−1^ [90,91] (see Figure 4b) and are used as biomarkers in antibiotic susceptibility assays. Drug sensitivity or resistance is detected by comparing the signal ratios at certain bands in the spectra between bacteria untreated and treated with antibiotics (r_Raman shift_) (Table 1). The differences between spectra are revealed within hours, which makes the Raman approach a faster method compared to conventional AST (antibiotic susceptibility tests) methods.

The obvious changes in SERS spectra of *E. coli* were revealed after 20 min of exposure to ampicillin, indicating the inhibition of microbial proliferation. When *S. aureus* was treated with cell wall targeting antibiotics such as ampicillin, vancomycin, and cefotaxime, the spectral changes were observed within an hour. Noticeably, the SERS spectrum of *S. aureus* after oxacillin treatment was temporarily restored, and the authors consider that the effect of the cell wall targeting antibiotic is compensated for by the thick layer of peptidoglycan found in Gram-positive bacteria. When treated with antibiotics that inhibit protein biosynthesis like gentamicin and tetracycline, the discernible SERS changes occurred only after 9–12 h [92].

Features in the spectra of amikacin-treated bacteria show that as the concentration of amikacin increases, the protein-related peaks of *P. aeruginosa* decrease, while the intensity of nucleic acid bands increases [93]. Several data analysis methods (principal component analysis, partial least squares, and 2D correlation analysis) link amikacin dose with the prominent protein-related peak at 1607 cm^−1^, apparently, because aminoglycosides inhibit protein biosynthesis.

The SERS signal of sensitive bacteria responds more dramatically to antibiotic treatment than that of their resistant counterparts [88,94]. The dependency of Raman shift intensity of antibiotic-susceptible species of *E. coli* via antibiotic concentration achieves the maximum, with the maximum intensity being achieved at the minimum inhibitory concentration (MIC), while the Raman signal of antibiotic resistant strain slightly depends on the antibiotic concentration [91].

Several SERS-based approaches, including the conventional drop method, extracellular matrix analysis, and filter mapping for investigating *E. coli O157:H7* susceptibility to ampicillin, were studied in [88]. Bacteria are differentiated by observing a peak, the intensity of which increases due to lysis of the bacterium by the antibiotic treatment and the release of intracellular purine compounds. All methods are effective in identifying susceptible or resistant bacteria based on bacterial response to antibiotic exposure. SERS can be performed on both bacterial culture and filtered extracellular matrix liquid. Matrix liquid analysis provides a more consistent SERS signal than the drop method, while SERS filter mapping gives a broader view of the variation within a bacterial sample.

Different concentrations of antibiotics influence the Raman spectra of bacteria treated with the antibiotics. The intensity of Raman spectra peaks at 735 cm^−1^ at MIC and 2×MIC concentrations were lower than that of untreated bacteria; in contrast, the highest intensity was observed at subMIC concentrations. This behavior was characteristic of *E. coli* treated with amikacin, ciprofloxacin, polymyxin, and tigecycline and *S. aureus* treated with ciprofloxacin, chloramphenicol, erythromycin, and vancomycin. Raman data were consistent with the standard medium method. The authors supposed that antibiotics at subMIC concentrations promoted bacteria reproduction, as revealed in the Raman spectra [90].

SERS also allows monitoring the development of bacterial resistance to the antibiotics by examining the changes in the signal intensity ratios characterizing molecular targets in bacteria. *S. aureus* resistance to oxacillin/cefazolin was detected based on the changes in I_734_/I_867,_ I_1163_/I_959_ and I_1372_/I_1349_ ratios [95]. I_734_/I_867_ showed a negative correlation with MIC values, while I_1163_/I_959_ and I_1372_/I_1349_ were increased with MIC values. When studying the development of *S. typhimurium* resistivity, the I_990_/I_1348_, I_1165_/I_1205_, and I_958_/I_1017_ ratios were monitored in dynamics [96]. In both studies, multivariate statistical analyses correctly differentiated strains with different resistance degrees.

### 2.5. Antibiotic Susceptibility Testing Using Raman Spectroscopy of Bacteria

As antibiotics are often employed to fight life-threatening infections, emerging antibiotic resistance demands rapid testing in the clinical conditions. Surface-enhanced Raman spectroscopy provides high sensitivity and robustness to the antibiotic susceptibility assays. Studies on antibiotic susceptibility testing are listed in Table 2.

Raman spectroscopy enables contactless and very fast (often in the range of seconds) signal acquisition, with a sensitivity down to single-cell level. With the appropriate signal-processing framework, this approach offers express study of the antibiotic–microbe interaction. We discuss below various the Raman-based techniques suitable for antibiotic susceptibility testing.

#### 2.5.1. Laser Tweezer–Assisted Normal Raman Spectroscopy

The ability to distinguish individual cells by their spectra is essential for the development of clinical diagnostic tools, as it allows rapidly detecting minor subpopulations in a heterogeneous population of cells. Laser-tweezer Raman spectroscopy combined with principal component analysis (PCA) has made it possible to identify various metabolic states of bacterial cells [97,106,107]. A significant difference was found in the behavior of *E. coli* cells in the presence or absence of cefazolin, penicillin, and streptomycin. Unexposed and treated *E. coli* cells form separate clusters, with minimal overlap between groups. Antibiotic-sensitive and -resistant strains of *S. mutans* were recognized as fast as in 30 min [105].

#### 2.5.2. Resonance Raman Spectroscopy

It was shown that UV resonance Raman spectroscopy can be used as a reliable tool for quantitative assay of the amikacin action against *P. aeruginosa* and agrees well with its presumed mode of action [93]. The clustering pattern in the space of discriminant factors directly correlated with the concentration of amikacin, and regression analysis by partial least squares (PLS) allowed predicting the concentration of the antibiotic that was exposed to bacterial cells. 2D-correlation spectroscopy showed that the spectral changes caused by the presence of amikacin were consistent with the presumed mode of amikacin action.

#### 2.5.3. Stimulated Raman Spectroscopy

Stimulated Raman scattering (SRC) metabolic imaging can be employed to visualize the glucose metabolic activity of living bacteria at the single-cell level [101]. Differences in the behavior of sensitive and resistant strains of *E. faecalis* were revealed this way. The MICs of vancomycin against *E. faecalis*, *E. coli*, *K. pneumoniae*, and *S. aureus* were determined and were two times higher than the MIC values obtained by the conventional cultured-based method. This discrepancy may be caused by the too-short incubation time (0.5 h) used in metabolic imaging. Mismatch in the inhibition of bacterial growth and inhibition of the metabolic activity could be another possible reason. Nevertheless, the antibiotic susceptibility defined at the single-cell level might be invaluable for studying non-cultured or fastidious bacteria.

Stimulated Raman scattering metabolic imaging allows measuring both metabolic activity and morphological changes in antibiotic-treated bacteria [111]. SRS and D_2_O labeling were applied to perform antibiotic susceptibility testing of cefotaxime on 103 *E. coli* strains. Metabolic activity was monitored using signals of C-D (carbon-deuterium) bonds of deuterated biomolecules. The morphological changes in bacteria were quantified based on the bacterium area of cefotaxime-susceptible/cefotaxime-resistant strains treated or not treated with antibiotics. Interestingly, C-D intensity or morphological deformation alone led to erroneous MIC estimation. Only the combination of signals from C-D and morphological deformation of bacteria was sufficient for MIC determination, with reported 93.2% essential agreement with the standard reference method.

#### 2.5.4. Surface-Enhanced Raman Spectroscopy

The reproduction of *S. aureus* and *E. coli* and their susceptibility to antibiotics can be quickly identified by studying the dynamic changes occurring in the single-cell SERS profiles on Ag/AAO substrates [92]. The response of *Lactococcus lactis* to antibiotics was observed in 60–90 min using SERS and PCA [98]. The distinct changes in SERS spectra allowed determining cell wall changes as well as the biochemical profile features. In another *S. aureus* and *E. coli* study, the changes in SERS spectra were evident after 120 min of antibiotic treatment [94]. SERS-based Gram classification and MIC estimation were consistent with a standard cultural method. It was found that MIC depended on the inoculum concentration, with a detection limit of about 10^5^ CFU/mL. The quantitative SERS assay with silver nanoparticles reported in [99] was suitable for urinary tract infections (correctly distinguishing samples containing more than 10^5^ CFU/mL, classifying bacteria pathogens with ~94% accuracy, and determining the antibiotic sensitivity of bacteria with 81–100% accuracy).

The study of the Raman spectra of sensitive and resistant *E. coli* cells in the presence of antibiotics revealed the coexistence of different spectral populations with ratios varying depending on the concentration of the antibiotic. The test procedure, overcoming single-cell heterogeneities, was devised to estimate the MIC and determinate the susceptibility phenotype of the tested bacteria using only a few single-cell spectra in 4 h, including the preculture step [104]. Isotope labeling with D_2_O allowed determination of the resistance of *E. coli, P. vulgaris, S. entérica, S. flexneri, K. variicola, E. fergusonii*, and *P. rettgeriprofiles* to ampicillin, chloramphenicol, kanamycin, and meropenem [110]. Spectral results obtained at the single-cell level were perfectly consistent with the standard disk-diffusion method. Isotope labeling was also used for the fast Raman-assisted antibiotic susceptibility test (FRAST) reported in [117]. FRAST and conventional AST were tested on six clinical strains (four Gram-negative and two Gram-positive) with 38 antibiotics and showed 88% agreement. For real clinical samples, FRAST results were consistent with the conventional AST and MALDI-TOF (Matrix-assisted laser desorption/ionization-time of flight) and allowed sample-to-report times as low as 3 h for urine and 21 h for blood samples.

A microwell method with SERS detection was successfully used for rapid antibiotic susceptibility tests. The microwell device can significantly reduce the required bacterial concentration for a detectable SERS signal. The bacterial LOD (limit of detection), while using the microwell platform, is much lower than conventional SERS-AST, and according to some reports, is 5 × 10^5^ [112] and 10^3^ CFU/mL [89] for *E. coli*. The microfluidic microwell device with an automated microfluidic control system as demonstrated in [91] can perform the automatic buffer washing procedure without significant detaching of bacteria from microwells. Moreover, antibiotics can be preloaded and vacuum-dried in the microfluidic device itself, enabling on-chip bacterial stimulation.

#### 2.5.5. DEP-Raman Spectroscopy

Manipulation of bacterial cells with dielectrophoresis (DEP) improves the identification, as it takes into account the dielectric properties of bacteria as well as their size and shape. The use of a combined DEP-Raman substrate significantly reduces the analysis time compared with the standard cultural method and reduces errors due to the differences in cultivation conditions and the standardization of differences between batches. Changes in the spectra occur in 90–120 min after the start of detection, making it possible to distinguish antibiotic-resistant strains from susceptible ones [100,102,103,113,115,121].

## 3. Prospects: Whole-Cell Biosensing of Antibiotics

Whole-cell biosensing provides numerous advantages over the traditional analytical chemistry methods and immunoassays. Bacteria are the natural target organisms for most antibiotics. Sensitive bacterial cultures can be easily maintained and scaled up, making them good candidates to be used in biosensing devices.

Bioluminescent, genetically modified *E. coli* cells were employed for tetracycline detection by Korpela et al. [122]. This approach was later developed into a bacterial reporter panel capable of detecting antibiotics from eight different structural classes, albeit with a rather low sensitivity [123].

A whole-cell multifunctional *E. coli* bacterial biosensor with a response reflecting the mode of action of the sensed antibiotic was demonstrated by Bianchi and Baneyx as early as 1999 [124]. Introduction of a β-galactosidase reporter gene fused with stress promoters (cold shock, cytoplasmic stress, or protein misfolding) provided the sensing of ribosome-targeting (chloramphenicol, tetracycline, streptomycin, neomycin), membrane-damaging (polymyxin B), and cell wall synthesis-inhibiting (carbenicillin) antibiotics. The biosensing was realized by the colorimetric assay or coculture method. A multifunctional bioluminescent biosensor was constructed in *Bacillus subtilis* and enabled the rapid screening of 14,000 natural products with a bactericidal action, elucidating the mode of action of several natural antibiotic candidates [125]. These biosensors can be supplemented by the fast-responding Raman detection, thus combining the multimodality of known antibiotic-sensing genetically modified bacteria with the rapid signal acquisition of Raman systems.

Despite the recent efforts in the direct SERS-based detection of antibiotics [25] and infectious bacteria [126], whole-cell SERS biosensing of antibiotics has not been systematically studied to the best of our knowledge and awaits further research.

## 4. Conclusions

The decades of antibiotic research have provided highly efficient bactericidal weapons aimed at specific prokaryotic targets: cell wall synthesis, cellular membrane, protein synthesis, and nucleic acids. However, the development of novel antibiotics is a costly and lengthy process, and for the foreseeable future, we will be limited in the choice of antibacterial drugs.

Widespread antibiotic pollution can pose a risk of multidrug resistance development in bacteria. Mechanisms of antibiotic resistance are known and sometimes circumvented by suitable inhibitors. However, rapid antibiotic susceptibility testing is still highly desirable in the clinical setting. Surface-enhanced Raman spectroscopy provides fast and contactless acquisition of antibiotic-induced changes in bacterial cells. Therefore, a SERS-based approach may serve as the analytical method for personalized medical treatment, especially when multidrug-resistant infection is suspected.

Finally, SERS-based monitoring of living bacterial cultures promises the creation of whole-cell biosensors for sensitive antibiotic detection. Interestingly, the same SERS setup may be further employed for direct the SERS detection of antibiotics, provided there is a suitable sample preparation kit. Thus, high sensitivity of bacterial sensors can be coupled with a high information capacity of SERS spectra.

## Figures and Tables

**Table 1:** Raman features observed in the antibiotic-treated bacteria.

Organism	Antibiotic Exposure	Raman Features *	Raman Technique	Reference
*E. coli*	Kanamycin 16–64 µg·mL^−1^ 2 h	r_740_ of susceptible bacteria decreases; r_740_ of resistant bacteria varies slightly	SERS	[89]
*E. coli*	Amikacin, Ciprofloxacin, Polymyxin, Tigecycline 2^−2^–2^−5^ µg·mL^−1^ 2 h	I_734_^2MIC^ < I_734_^MIC^ < I_734_^control^ < I_734_^0.5MIC^ < I_734_^0.25MIC^	SERS	[90]
*S. aureus*	Ciprofloxacin, Chloramphenicol, Erythromycin, Vancomycin 2^−2^–2^−5^ µg·mL^−1^ 2 h			
*E. coli*	Ampicillin 4–32 µg·mL^−1^ 3 h	I_733_, r_733_ of susceptible bacteria increase until the MIC is reached, and then decrease; I_733_, r_733_ of resistant bacteria vary slightly;	SERS	[91]
*E. coli*	Ampicillin 20 µg·mL^−1^ (5MIC) Up to 3 h; first features revealed after 20 min of antibiotic exposure	I_725_, I_1095_ decrease, which is accompanied by new SERS peak appearance over 90 min	SERS	[92]
*S. aureus*	Oxacillin 5 µg·mL^−1^ (5MIC) Up to 3 h; first features revealed after 50 min of antibiotic exposure	Appearance of new peaks at 50 min, sharp decline of I_732_, 10–20 min later, newly formed peaks disappeared, I_732_ recovery, 732 peak disappearance after 120–180 min		
	Gentamicin, Tetracycline Up to 12 h	Characteristic SERS response I_732_ was not noted until after 9–13 h of treatment		
*P. aeruginosa*	Amikacin 0.25–6 µg·mL^−1^ Overnight incubation	I_1607_ decreases	UV resonance Raman	[93]
*S. aureus*	Oxacillin, Vancomycin 0.5–2 µg·mL^−1^ Up to 6 h for Oxacillin; Up to 2 h for Vancomycin; first features revealed within one hour	r_730_ decreases; r_730_ of resistant bacteria varies slightly	SERS	[94]
*E. coli*	Imipenem 0.03–012 µg·mL^−1^ Up to 6 h; first features revealed within one hour	r_654_, r_724_ decrease; r_654_, r_724_ of resistant bacteria vary slightly	SERS	
*E. coli*	Ampicillin 1 mg·mL^−1^ (excess) 3 h of incubation	I_733_ of susceptible bacteria increases; I_733_ of resistant bacteria varies slightly	SERS	[88]
*S. aureus*	Oxacillin, Cefazolin 0.125–32 µg·mL^−1^ 21 days of exposure	I_734_/I_867_ decreases, I_1372_/I_1349_, I_1163_/I_959_ increase as antibiotic resistance develops	SERS	[95]
*Salmonella typhimurium*	Cefotaxime 0.5–4 µg·mL^−1^ 50 days of exposure	I_990_/I_1348_ increases, I_1165_/I_1205_, I_958_/I_1017_ decrease as antibiotic resistance develops	SERS	[96]

* I—intensity of Raman signal, r—the ratio of Raman signal intensities at a certain Raman shift.

**Table 2:** Antibiotic susceptibility studied by Raman spectroscopy.

Antibiotic	Target Organism	Raman Protocol	Reference
Ampicillin, Vancomycin, Cephotaxim, Oxacillin, Gentamicin, Tetracycline	*S. aureus, E. coli*	SERS; Raman microscope; Excitation at 632.8 nm; 10^5^ W × cm^−2^; Ag/AAO substrate	[92]
Amikacin	*P. aeruginosa*	RRS; Raman spectrometer; Excitation at 244 nm; 0.5 mW; CaF_2_ substrate	[93]
Penicillin/streptomycin	*E. coli*	NR; laser tweezer Raman microscope; Excitation at 785 nm; 28 mW; Cell chamber with a fused-silica microscope coverslip	[97]
Ampicillin, Ciprofloxacin	*L. lactis*	SERS; Raman microscope; Excitation at 780 nm; 1 mW; 50 nm citrate-capped AgNPs on a gold-stained glass slide substrate	[98]
Oxacillin, Imipenem, Vancomycin	*S. aureus, E. coli*, *A. baumannii, K. pneumoniae*	SERS; Raman microscope; Excitation at 632.8 nm; 10^5^ mW × cm^−2^; two-dimensional hexagonally packed AgNPs embedded in nanochannels of anodic aluminum oxide substrate	[94]
Augmentin, Amoxicillin, Cefaclor, Cefuroxime, Cefazolin, Ceftriaxone, Ciprofloxacin	*Proteus sp., K. pneumoniae, E. coli*	SERS; portable Raman spectrometer; Excitation at 532 nm; 50 mW; 100 μm AgNPs substrate	[99]
Ciprofloxacin	*E. coli*	NR; Raman microscope; Excitation at 532 nm; 36 mW; DEP (dielectrophoresis)-based microfluidic device	[100]
Vancomycin, Linezolid, Daptomycin, Gentamicin, Erythromycin	*E. faecalis, E. coli, K. pneumoniae, S. aureus*	SRS; custom-built dual-laser Raman microscope; Excitation at 847 + 1040 nm; Agar gel pad on coverglass	[101]
Ciprofloxacin	*E. coli*	NR; confocal Raman microscope; Excitation at 532 nm; 15 mW; DEP (dielectrophoresis) chip	[102]
Vancomycin	*E. faecalis, E. faecium*	NR; confocal Raman microscope; Excitation at 532 nm; 15 mW DEP (dielectrophoresis) chip	[103]
Gentamicin, Ciprofloxacin, Amoxicillin	*E. coli*	NR; confocal Raman microscope; Excitation at 532 nm; 9 mW; Coverslip substrate	[104]
Ampicillin	*S. mutans*	NR; Raman microscope; Excitation at 532 nm; 3–5 mW; CaF_2_ substrate	[105]
Cefazolin	*E. coli*	NR; laser tweezer Raman microscope; Excitation at 785 nm; 28 mW; Cell chamber with a fused-silica microscope coverslip	[106]
Penicillin, G-streptomycin, Cefazolin	*E. coli*	NR; laser tweezer Raman microscope; Excitation at 785 nm; 28 mW; Cell chamber with a fused-silica microscope coverslip	[107]
Cefotaxime	*E. coli*	NR; laser tweezer Raman microscope; Excitation at 785 nm, 532 nm; 150 mW; Microfluidic chip	[108]
Oxacillin, Imipenem, Methicillin	*S. aureus, A. baumannii, P. aeruginosa*	SERS; portable Raman spectrometer; Excitation at 785 nm; 25 mW; AgNPs SERS substrate	[109]
Kanamycin	*E. coli*	SERS; Raman microscope; Excitation at 632.8 nm; 5 mW; Silver island film sputtered substrate in a microfluidic system	[89]
Ampicillin, Chloramphenicol, Kanamycin, Meropenem	*E. coli, P. vulgaris, S. entérica, S. flexneri,* *K. variicola, E. fergusonii, P. rettgeri*	NR; confocal Raman microscope; Excitation at 532 nm; D_2_O-labeling	[110]
Cefotaxime	*E. coli*	SRS; custom-built dual-laser Raman microscope; Excitation at 852 nm (~20 mW) + 1045 nm (~300 mW); Coverslip substrate	[111]
Kanamycin	*E. coli*	SERS; Raman microscope; Excitation at 632.8 nm; 50 mW; Microfluidic microwell device AgNP@AAO substrate	[112]
Ciprofloxacin, Cefotaxime, Piperacillin	*E. coli*	NR; confocal Raman microscope; Excitation at 532 nm; DEP setup	[113]
Ciprofloxacin	*B. pumilus*	RRS; Raman microscope; Excitation at 244 nm; 32 mW	[114]
Amikacin, Ciprofloxacin, Polymyxin B, Tigecycline, Ciprofloxacin, Chloramphenicol, Erythromycin, Vancomycin	*E. coli, S. aureus*	SERS; Raman microscope; Excitation at 532 nm; 14 mW; Bacteria-aptamer@AgNPs substrate	[90]
Ciprofloxacin	*E. coli*	NR; confocal Raman microscope; Excitation at 532 nm; 10 mW; DEP microfluidic device	[115]
Ampicillin	*E. coli*	SERS; confocal Raman microscope; Excitation at 785 nm; 20 mW; Au@AgNR tag substrate	[116]
Ampicillin	*E. coli*	SERS; Raman microscope; Excitation at 632.8 nm; 5 mW; Microfluidic microwell device substrate	[91]
Amikacin, Azithromycin, Aztreonam, Cefazolin, Cefepime, Cefmetazole Na, Cefoperazone/sulbactam, Cefoxitin, Ceftazidime, Ceftazidime/avibactam, Ceftolozane/tazobactam, Ceftriaxone, Cefuroxime, Ciprofloxacin, Clindamycin, Doxycycline, Ertapenem, Erythromycin, Gentamicin, Imipenem, Levofloxacin, Linezolid, Meropenem, Minocycline, Moxifloxacin, Nitrofurantoin, Nitrofurantoin, Oxacillin, Penicillin, Piperacillin, Piperacillin/tazobactam, Polymyxin, Rifampicin, Teicoplanin, Tetracycline, Ticarcillin/clavulanic acid, Tigecycline, Tobramycin, Tobramycin, Trimethoprim−sulfamethoxazole, Vancomycin	*E. coli, P. aeruginosa, K. pneumoniae, E. faecium, S. aureus, S. epidermidis, S. hominis*	NR; confocal Raman microscope; Excitation at 532 nm; D_2_O-labeling; Aluminum-coated slide substrate	[117]
Ampicillin	*E. coli*	SERS; confocal Raman microscope; Excitation at 780 nm; 4 mW; 55 nm AuNPs substrate	[88]
Oxacillin, Cefazolin	*S. aureus*	SERS; portable Raman spectrometer; Excitation at 785 nm; 200 mW; 50 nm AuNPs substrate	[95]
Cefotaxime	*S. typhimurium*	SERS; portable Raman spectrometer; Excitation at 785 nm; 200 mW; 40–60 nm AuNPs substrate	[96]
Ampicillin, Neomycin, Chlortetracycline	*Escherichia coli, Bacillus cereus, Salmonella enterica*	SERS; portable Raman spectrometer; Excitation wavelength not reported; 55 nm AuNPs substrate	[118]
Minocycline, Levofloxacin	*Elizabethkingia* spp.	NR; confocal Raman microscope; Excitation wavelength not reported; 5 mW; Aluminum-coated slide substrate	[119]
Oxacillin, Cefotaxime	*S. aureus* *E. coli*	SERS; Raman microscope; Excitation at 632.8 nm 10^5^ Mw × cm^−2^; AgNPs array embedded in nanochannels of anodic aluminum oxide substrate	[120]

## Data Availability

Not applicable.
